# Double defense: enhancing tobacco with cyanobacterial and thaumatin genes

**DOI:** 10.3389/fbioe.2026.1667530

**Published:** 2026-01-16

**Authors:** Tetiana Kyrpa, Yelizaveta Prokhorova, Maksym Kharkhota, Mykola Kuchuk

**Affiliations:** 1 Institute of Cell Biology and Genetic Engineering National Academy of Sciences of Ukraine, Kyiv, Ukraine; 2 Danylo Zabolotny Institute of Microbiology and Virology National Academy of Sciences of Ukraine, Kyiv, Ukraine

**Keywords:** climate change, desaturases, plant stress, thaumatin, tobacco

## Introduction

Global climate change exacerbates both abiotic and biotic stress factors, which affect not only agricultural crops but also native plant species that must adapt to rapidly changing ecosystems (Raza et al., 2025). One of the universal mechanisms of plant resistance to abiotic factors is an increase in the proportion of unsaturated fatty acids in membrane phospholipids (Xiao et al., 2022). Desaturases are enzymes that facilitate the formation of double bonds in fatty acids, transforming them from saturated to unsaturated (Ferrero et al., 2025). This increase in unsaturated fatty acid content enhances membrane plasticity and viscosity while lowering the crystallization temperature, thereby improving plant resilience against a range of abiotic stressors, including low temperatures, frost, and drought (Yu et al., 2021).

In addition to abiotic stress, the physiological health and productivity of plants hinge on various factors, including susceptibility to fungal and viral pathogens, which can hinder plant development, disrupt photosynthesis, and impair essential biochemical processes (Yang and Luo, 2021). Interestingly, abiotic stressors can sometimes create favourable conditions for pathogen development (Ortel et al., 2024). Some of the plants defence mechanisms are aimed at preventing pathogen entry, while others inhibit the progression of infections within the plant (El-Saadony et al., 2022). In many plant species, Thaumatin-Like Proteins (TLPs), which are structurally related to thaumatin II, are prevalent (Zhao et al., 2024), and show antifungal activity based on their capacity to disrupt the cell walls of pathogenic fungi (Osei-Obeng et al., 2024). These proteins have β-1,3-glucanase activity and can bind to degrade β-1,3-glucan, a fundamental component of fungal cell walls, facilitating further membrane destruction (Liu et al., 2021). Moreover, thaumatins may influence the activity of other proteins through metabolic pathway regulation (Zhao et al., 2024).

In this study, we introduced the cyanobacterium Δ-12-acyl-lipid desaturase gene (*des*A) from *Synechocystis* sp. PCC 6803 and the thaumatin II gene, (thII) from *Thaumatococcus daniellii* into the *Nicotiana tabacum* genome. The presence of a transgene in a plant can yield both advantageous and detrimental outcomes, as gene expression and protein functionality require additional resources, potentially leading to biochemical and physiological competition. Therefore, the *des*A gene was placed in a vector regulated by the cold-inducible CBF1 promoter from *Arabidopsis thaliana*. This study aimed to explore the concurrent functioning of two proteins with different substrate specificities, would exhibit augmented resistance to both abiotic and biotic stressors.

## Materials and methods

### Genetic constructs

This research used cloned sequences of the *des*A gene, which encodes the Δ12-acyl-lipid desaturase from *Synechocystis* sp. PCC 6803, and the *thII* gene from *T. daniellii,* which encodes a sweet-tasting thaumatin II protein structurally related to the TLP family. The desaturase genes were translationally fused to the *lic*BM3 gene, which encodes a reporter protein derived from the thermostable lichenase of *Clostridium thermocellum*. The hybrid genes, *des*A:*lic*BM3, were cloned into pBISN-based vectors containing the selectable *npt*II gene, regulated by the cold-induced promoter CBF1 from the *A. thaliana*. The thaumatin II gene was integrated into a pNMD46732-based vector, with a selectable *bar*, under the control of the constitutive 35S cauliflower mosaic virus (CaMV) DNA promoter.

Control groups included transgenic plants with vector constructs incorporating the GFP reporter gene instead of the desaturase gene (*des*A), as well as wild-type tobacco (*N. tabacum*). All genetic constructs and transgenic plants were sourced from the collection at the Institute of Cell Biology and Genetic Engineering.

### Agrobacterium tumefaciens-mediated plant transformation

Leaf blades measuring 1–1.5 cm^2^ were excised and placed into a bacterial suspension, which was incubated for 1 hour at +25 °C in the dark. The explants were then collected, washed to remove excess bacterial culture, and transferred to MS medium. They were co-cultivated with *Agrobacterium* for 2 days at +25 °C (to promote *Agrobacterium* growth). After co-cultivation, the explants were washed in sterile distilled water, dried on sterile filter paper for 10–15 min, and then placed in MS medium supplemented with the phytohormones BAP (1 μg mL^-1^) and NAA (0.1 μg mL^-1^), along with 700 μg mL^-1^ cefotaxime to inhibit *Agrobacterium* growth. Regeneration of potential transgenic shoots was monitored over a period of 2–3 weeks, with regeneration occurring *in vitro* at 25 °C ± 3 °C under a 16-h photoperiod with light intensity of 100 μmol quanta (m^2^s).

### Conditions for the polymerase chain reaction

Plant DNA was isolated using a standard CTAB method (Mark et al., 2024), and its concentration was determined by measuring optical density at 260 nm with a BioPhotometer spectrophotometer (Eppendorf, Germany). PCR was conducted using a 2720 Thermal Cycler (Applied Biosystems, USA), with primers *th*II-f (cac​ctt​cga​gat​cgt​caa​ccg​ctg) and *th*II-r (aag​ctt​agg​cag​tag​ggc​aga​aag​tg). Amplification conditions included 5 min at 94 °C; 30 cycles of 30 s at 94 °C, 45 s at 63 °C, and 45 s at 72 °C; followed by 5 min at 63 °C. Amplification products were separated via 1% agarose gel electrophoresis using TAE buffer and visualized with ethidium bromide. The O’GeneRuler 1 kb DNA Ladder (Fermentas, Lithuania) served as a DNA marker.

### Qualitative determination of thermostable lichenase activity

The preparation of plant material and the reaction were carried out according to the protocol described earlier (Gerasymenko et al., 2015). A qualitative lichenase assay and part of analysis of the FA was conducted 40 min after exposure to cold stress (0 °C for 30 min, followed by −5 °C for 80 min).

### Analysis of the fatty acid spectrum by gas chromatography and mass spectrometry

Samples for gas chromatography and mass spectrometry analysis were prepared according to previously published protocols (Gerasymenko et al., 2015). The analysis was conducted using an Agilent 6890N/5973 inert chromatographic-mass spectrometry system equipped with a DBFFAP capillary column (J&W Scientific, United States). Fatty acid methyl esters (FAMEs) were identified by comparing the obtained spectra with entries from the NIST 02 mass spectrum library and standard mixtures of bacterial FAMEs (47080U, Supelco).

For fatty acid analysis, mixed plant material from three lines of *N. tabacum* CBF1:*des*A:*lic*BM3+*th*II, three lines of *N. tabacum* CBF1:*des*A:*lic*BM3, two lines of *N. tabacum* CBF1:GFP:*lic*BM3 obtained from transgenic plants, and *N. tabacum* were used. Six biological replicates and one analytical replicate were selected (upper unfolded leaves). The statistical significance of differences between average means was estimated using Student’s paired *t-*test, *p*-values were calculated using Excel standard functions.

### Visualizations

**FIGURE 1:**
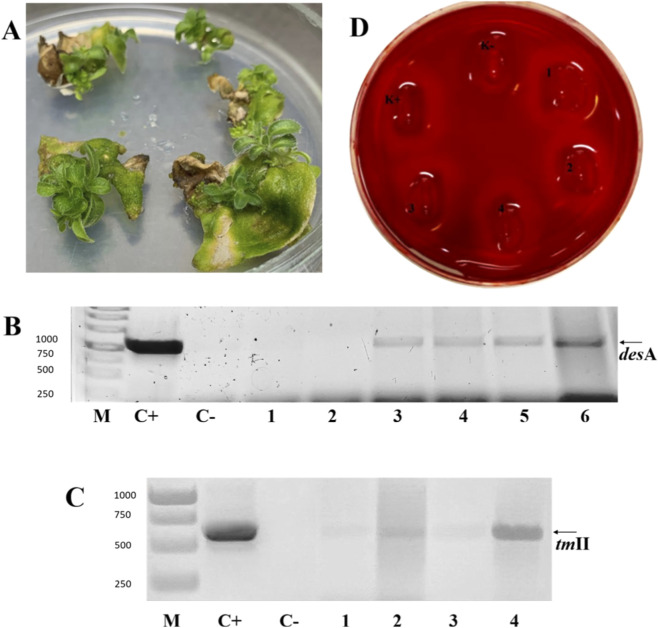
**(A)** Tobacco plant regeneration after genetic transformation. **(B)** PCR analysis of the genomic DNA of transgenic tobacco for *des*A insert. М - DNA mass ladder O`GeneRuler™ 1 kb DNA Ladder (Thermo Fischer Scientific); C + - positive control, pNPB14 plasmid DNA, C- - negative control, non-transformed plant, 1-6 – tested transformed tobacco (PCR-product - 949 bp). **(C)** PCR analysis for the presence of the *th*II gene insert: M - DNA mass ladder, C + - positive control, pNMD46732 plasmid DNA, C- - negative control, one to four total DNA from tobacco plant lines (PCR-product - 626 bp). **(D)** Qualitative test for lichenase activity: K + – positive control, *Nicotiana tabacum* expressing *lic*BM3 gene, K- – negative control, non-transgenic plant; 1-4 – tobacco plant extract simultaneously expressing *des*A and *th*II.

The genetic transformation of transgenic *N. tabacum*, which demonstrated the insertion and expression of the *des*A gene, was carried out using a plasmid vector containing the *th*II gene. The active regeneration phase of the plants was observed within 1–1.2 months (Figure 1A). Two months post-transformation, regenerants were cultured on standard MS medium. After biomass growth, the presence of the *th*II gene insertion was confirmed via PCR (Figure 1B).

The expression of the desaturase gene was indirectly assessed through the activity of the thermostable lichenase reporter protein (*lic*BM3), since both the desaturase and lichenase genes are located within the same reading frame under the control of the cold-inducible promoter. The assay evaluates the lichenase’s ability to degrade the complex carbohydrate lichenan (Figure 1C). The number of replicates for gene insertion detection and lichenase activity testing was 3, all of which were positive.

Results indicated that the plants maintained lichenase activity, suggesting the continued expression of the *des*A gene and lichenase activity. Tobacco plants demonstrating confirmed insertion and expression of the 12-acyl-lipid desaturase genes exhibited heightened linoleic acid content. To compare fatty acid spectra, tobacco plants with an insertion both the *des*A and *th*II genes, those only the *des*A gene, those with the GFP:*lic*BM3 gene, and wild-type tobacco plants were analyzed. The upper open leaves were used for this analysis, which was performed under normal physiological conditions and following cold stress exposure. An increase in linoleic acid (C18:2) proportion was observed in plants with the *des*A gene insertion, differing from the fatty acid spectrum analysis results of the transgenic control and wild-type tobacco plants. No significant distinctions in fatty acid composition between plants with the desaturase gene and those with both desaturase and thaumatin gene insertions were observed (Table 1).

**TABLE 1:** Fatty acid composition (%).

Plants	Condition	С16:0	С16:1	С18:0	С18:2	С18:3
Control *N. tabacum*	Normal	21.3 ± 0.5	1.5 ± 0.9	1.1 ± 0.5	18.7 ± 0.6	56.9 ± 6.1
	Cold	20.8 ± 0.8	1.6 ± 0.8	1.3 ± 0.8	18.5 ± 5.3	57.5 ± 5.7
Control *N.tabacum* (СВF1:GFP:*lic*BM3)	Normal	21.2 ± 1.6	1.6 ± 0.5	1.2 ± 0.4	18.8 ± 4.3	55.6 ± 3.5
	Cold	20.9 ± 2.6	1.7 ± 0.6	1.8 ± 0.3	17.5 ± 4.7	57.9 ± 3.2
*N.tabacum* (СВF1:*des*A:*lic*BM3)	Normal	18.9 ± 0.5	1.3 ± 0.5	1.7 ± 0.4	24.3 ± 3.8	53.2 ± 5.3
	Cold	19.5 ± 0.6	1.3 ± 0.4	3.1 ± 0.6	33.3* ± 7.9	59.3 ± 4.8
*N.tabacum* (СВF1:*des*A:*lic*BM3+ *th*II)	Normal	19.1 ± 0.6	1.2 ± 0.4	1.9 ± 0.3	24.5 ± 3.7	51.4 ± 4.7
	Cold	20.3 ± 1	1.1 ± 0.4	2.5 ± 0.5	28.9* ±6.8	59.8 ± 4.6

## Contextualization

Developing biotechnological plants with transgenes that enhance resistance to various stress types represents a highly promising area of research (Ricroch et al., 2022). Currently, several biotechnological methods are employed to create new plant varieties via molecular breeding techniques (Sun et al., 2024). However, the effects of transgenes within plant organisms remain an inadequately explored subject (Fenzi et al., 2024). The influence of desaturase transgene expression on plant resistance to various abiotic factors is well-documented in many studies (Kumari et al., 2025), primarily because the proteins encoded by this gene have binding substrates within the plant due to their phylogenetic similarity to plant desaturases (Laureano et al., 2021). Similarly, thaumatin II protein is functionally relevant to plants (Hu et al., 2025). While several reports analyze and study the expression of specific transgenes in plants, few address the simultaneous insertion and expression of multiple transgenes with distinct substrate specificities. This paper presents preliminary findings on the generation of double transformants in tobacco plants, confirming the insertion of two different transgenes: the cyanobacterial desaturase and thaumatin II. However, the expression and pleiotropic effects of certain transgenes under stressful conditions may negatively affect or suppress the functionality of others (Cabrera et al., 2022), performing the functions of less stable proteins. This study aimed to elucidate how the expression of the *des*A gene, encoding Δ12-acyl-lipid desaturase from *Synechocystis* sp. PCC 6803, and the insertion of the *th*II gene from *Thaumatococcus daniellii* influence the function of these genes. TLPs are known to stabilize under extreme thermal and pH conditions due to their disulfide bridge structures, as well as resist protein degradation, potentially impacting the functionality of other proteins (Feng et al., 2024). So far, we have observed sustained expression of the desaturase gene and the activity of the target protein, with subsequent steps aimed at detecting the expression of the thaumatin gene and examining the plants' responses under stress conditions.

## Conclusion

The conducted experiments to create plants exhibiting enhanced resistance to abiotic and biotic stresses yielded potentially successful outcomes. For the first time, the simultaneous insertion of the taumatine II gene into tobacco was studied, which did not interfere with the expression and activity of the desaturase enzyme associated with the *des*A gene. Current efforts are focused on testing and confirming the expression systems for the thaumatin gene, with further studies planned to assess the resistance of the double transformants to low-temperature stress and pathogenic threats.

## Data Availability

The original contributions presented in the study are included in the article/supplementary material, further inquiries can be directed to the corresponding author.
